# Supramodal neural networks support top‐down processing of social signals

**DOI:** 10.1002/hbm.25252

**Published:** 2020-10-19

**Authors:** Melina Sonderfeld, Klaus Mathiak, Gianna S. Häring, Sarah Schmidt, Ute Habel, Raquel Gur, Martin Klasen

**Affiliations:** ^1^ Department of Psychiatry, Psychotherapy, and Psychosomatics, Medical School RWTH Aachen Aachen Germany; ^2^ JARA‐Translational Brain Medicine RWTH Aachen University Aachen Germany; ^3^ Life & Brain ‐ Institute for Experimental Epileptology and Cognition Research Bonn Germany; ^4^ Department of Psychiatry, Perelman School of Medicine University of Pennsylvania Philadelphia Pennsylvania USA; ^5^ Interdisciplinary Training Centre for Medical Education and Patient Safety ‐ AIXTRA, Medical Faculty RWTH Aachen University Aachen Germany

**Keywords:** emotion, gender, salience network, social perception, supramodal, top‐down

## Abstract

The perception of facial and vocal stimuli is driven by sensory input and cognitive top‐down influences. Important top‐down influences are attentional focus and supramodal social memory representations. The present study investigated the neural networks underlying these top‐down processes and their role in social stimulus classification. In a neuroimaging study with 45 healthy participants, we employed a social adaptation of the Implicit Association Test. Attentional focus was modified via the classification task, which compared two domains of social perception (emotion and gender), using the exactly same stimulus set. Supramodal memory representations were addressed via congruency of the target categories for the classification of auditory and visual social stimuli (voices and faces). Functional magnetic resonance imaging identified attention‐specific and supramodal networks. Emotion classification networks included bilateral anterior insula, pre‐supplementary motor area, and right inferior frontal gyrus. They were pure attention‐driven and independent from stimulus modality or congruency of the target concepts. No neural contribution of supramodal memory representations could be revealed for emotion classification. In contrast, gender classification relied on supramodal memory representations in rostral anterior cingulate and ventromedial prefrontal cortices. In summary, different domains of social perception involve different top‐down processes which take place in clearly distinguishable neural networks.

## Graphical abstract

The perception of facial and vocal stimuli is driven by cognitive top‐down influences. In the present study, we identified specific neural networks for the contributions of attentional focus and supramodal memory, depending on the classification task.

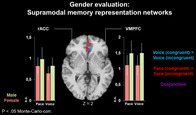

## 1 INTRODUCTION

The social evaluation of another person is based on visual and auditory signals, such as facial and vocal cues (Hensel, Bzdok, Müller, Zilles, & Eickhoff, 2015; Klasen, Chen, & Mathiak, 2012; Klasen, Kenworthy, Mathiak, Kircher, & Mathiak, 2011; Massaro & Egan, 1996). Social perception encompasses different kinds of information about our counterpart, including variable conditions such as the current affective state, but also fixed characteristics such as gender (Joassin, Maurage, & Campanella, 2011), up to complex social judgments about traits (Adolphs, 2003). Thus, social perception has great influence on our behavior toward others (Alcalá‐López et al., 2018; Bzdok et al., 2012; Hughes, Dispenza, & Gallup, 2004; Todorov, 2008). It is well established that the perception of auditory and visual social stimuli is driven not only by physical stimulus properties, but also by top‐down processes (Gilbert & Li, 2013; Latinus, VanRullen, & Taylor, 2010). “Top‐down processes” is a collective term for various types of cognitive influences driving perception. Important top‐down influences on perception are attentional focus, that is, the aspect of a stimulus that a person is attending to (Corbetta & Shulman, 2002; Hopfinger, Buonocore, & Mangun, 2000; van Atteveldt, Formisano, Goebel, & Blomert, 2007) or supramodal representations in long‐term memory (Choi, Lee, & Lee, 2018; Ramsey, Cross, & Hamilton, 2013). Some previous studies have addressed the role of specific top‐down contributions in social perception. Bzdok et al. (2012) separated neural networks underlying social, face‐specific, emotional and cognitive stimulus processing aspects. These findings suggest that there are neural networks that are driven by top‐down influences such as the task, but not by the stimulus material itself. Further evidence for this notion comes from a study by Hensel et al. (2015), who identified an involvement of dorsomedial prefrontal cortex (DLPFC) specifically during social trait judgments irrespective from stimulus modality.

Following the line of these studies, the present study investigated the neural networks underlying two types of top‐down influences on the perception of voices and faces: attentional focus (i.e., the attended aspect of the stimulus material) and memory representations. Attentional focus was varied via the task, that is, attending to either emotion or gender of the faces and voices. Moreover, social evaluation requires a comparison with a representation in the individual's long‐term (or “reference”) memory (Roitblat, 1987), which has been formed via previous experience (Mazur, 2017). For social evaluation, we were interested if the respective networks were supramodal, that is, independent from stimulus modality. To identify such supramodal memory representations, we developed the Social Implicit Association Test (SIAT), a social variant of the well‐established Implicit Association Test (IAT; Greenwald, McGhee, & Schwartz, 1998). The SIAT is described in detail in the Section 2; in short, it investigates associations between memory representations via reaction times to the respective stimuli. In the original IAT, associated stimuli (such as the words “doctor” and “hospital”) lead to faster responses than non‐associated stimuli (such as “bird” and “cigarette”; cf. Collins & Loftus, 1975). In the SIAT, we similarly assumed supramodal associations between the same social categories in voice and face, for example, between a happy face and a happy voice. From a neurobiological perspective, we assumed that such an association may be reflected by a shared brain region. In other words, we assumed that associated representations are in fact two aspects of one and the same concept and represented in the same brain region. With respect to faces and voices, this would correspond to a supramodal memory representation. There are previous functional magnetic resonance imaging (fMRI) studies on the IAT following the same logic. As an example, Knutson, Mah, Manly, and Grafman (2007) used the IAT during fMRI to investigate the neural substrates of gender and racial bias, identifying ventromedial prefrontal cortex (VMPFC) and ventral anterior cingulate cortex (vACC) as putative regions. These findings are well in line with Milne and Grafman (2001), who found a reduced IAT effect in patients with VMPFC lesions.

Based on these assumptions, we derived the following hypotheses:

1. For both emotion and gender evaluation, we can identify networks that are driven by attention: specific for the task (emotion/gender), but independent from stimulus modality or memory representations.
2. For both emotion and gender evaluation, we can identify networks that are driven by supramodal memory representations: independent from stimulus modality, but only present for associated auditory and visual stimuli.

These hypotheses were tested using fMRI.

## 2 MATERIALS AND METHODS

### 2.1 Participants

Forty‐five right‐handed subjects (23 female; age span 19–33 years, mean 24.7 ± 3.1) participated in the experiment. All subjects had normal or corrected to normal vision, normal hearing, no contraindications against MR investigations, and no history of neurological or psychiatric illness. All participants had either German as a first language or were grown up bilingually (with German from early childhood on).

The experiment was designed according to the Code of Ethics of the World Medical Association (Declaration of Helsinki, 2013, and the study protocol was approved by the Ethics Committee of the Medical Faculty at RWTH Aachen University (EK 003/14). After complete description of the study to the subjects, written informed consent was obtained.

### 2.2 Stimuli

Auditory stimuli were disyllabic pseudowords (Thonnessen et al., 2010). They followed German phonological rules but had no semantic content and were validated in a pre‐study on 25 subjects who did not participate in the fMRI study (see Klasen et al., 2011 for details of stimulus validation). Auditory stimulus duration was 1 s. Visual stimuli were taken from the validated NimStim Face Stimulus Set (Tottenham et al., 2009). In analogy to the duration of auditory stimuli, photographs were presented for 1 s each. Stimuli were always presented in isolation (unimodal presentation). Auditory and visual stimuli were counterbalanced for emotion (50% happy, 50% angry) and gender of the speaker/actor (50% female, 50% male). Moreover, each stimulus type was displayed by four different speakers/actors. In summary, the experiment thus comprised 32 different stimuli: 2 modalities (auditory/visual) × 2 emotions (happy/angry) × 2 genders (female/male) × 4 actors/speakers.

### 2.3 Experimental design

In the present fMRI study, we employed a SIAT. The SIAT measures crossmodal associations between corresponding visual and auditory modalities of social signals (faces and voices) via reaction times. Similar to the original IAT, the SIAT uses a classification task with two target categories sharing one response key, paired in either congruent or incongruent fashion. In the congruent condition, corresponding visual and auditory signals (e.g., happy faces and happy voices) share the same response key, whereas in the incongruent condition non‐matching pairings (e.g., happy faces and angry voices) are mapped on the same key.

To address the top‐down influence of attentional focus on social perception, two different SIAT variants were employed: an emotion SIAT, and a gender SIAT. Attentional focus was varied via the task. In the Emotion SIAT, the task was to classify the emotion of faces and voices (happy or angry), and in the Gender SIAT, the task was to classify stimulus gender (male or female). The task of the participant was to classify the stimuli according to the respective instruction as fast and as accurately as possible by pressing one of two response keys according to the assigned category. Pairings of target categories were either congruent or incongruent. In the congruent condition, corresponding auditory and visual stimuli were always mapped on the same key, for example, for emotion, angry voice and angry face on one key and happy face and happy voice on the other key. In the incongruent condition, non‐corresponding auditory and visual stimuli were mapped on the same key, for example, for emotion angry face and happy voice on one key and happy face and angry voice on the other key. The gender SIAT was designed in analogy.

**FIGURE 1:**
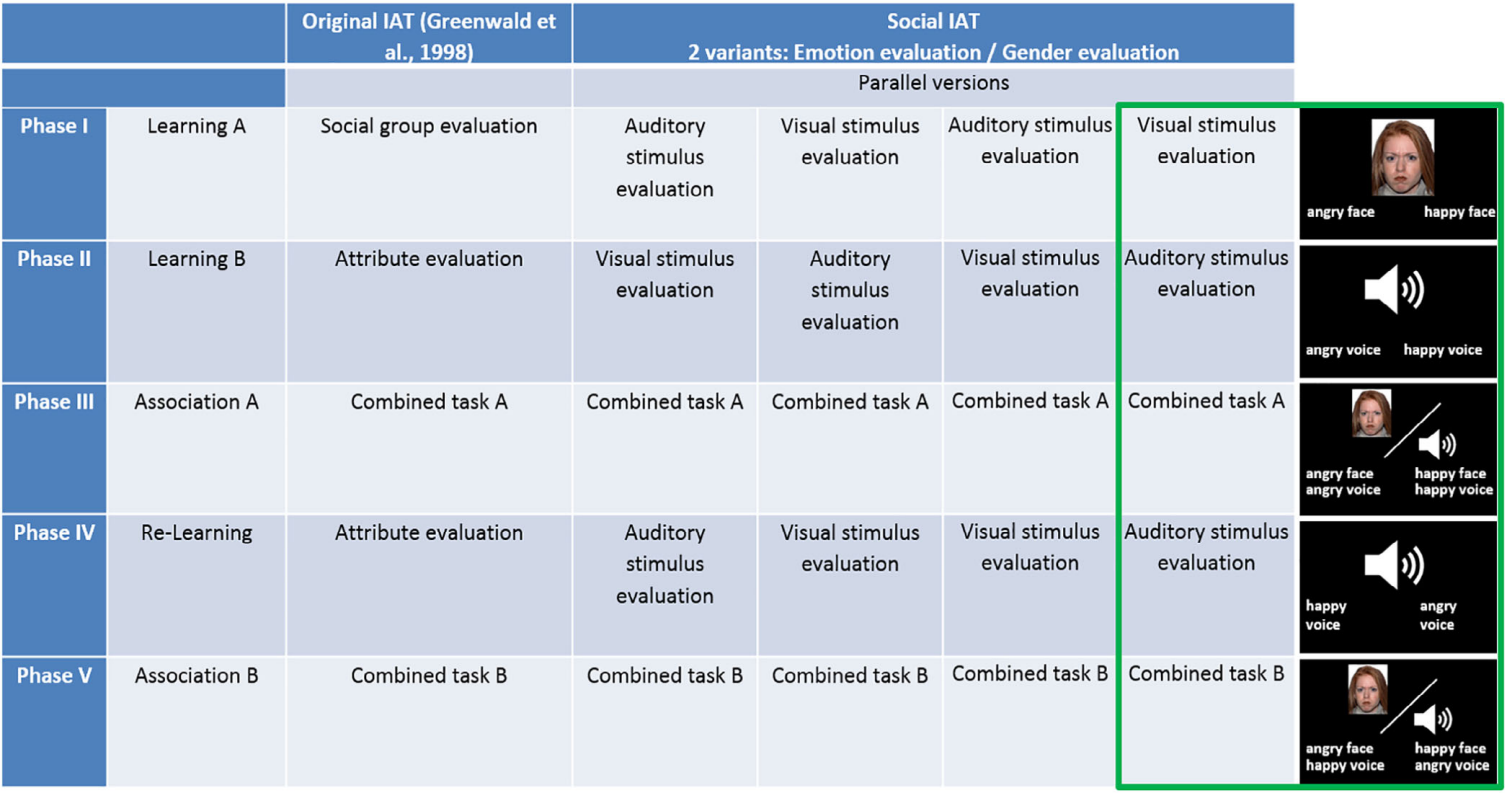
Experimental design. Two Social Implicit Association Test (SIAT) variants were employed: one for emotion evaluation and one for gender evaluation. Both SIATs consisted of five phases, in close analogy to the original IAT by Greenwald et al. (1998). To avoid sequential effects of auditory and visual evaluation phases, four parallel versions were employed for each of the SIATs (emotion/gender). Inserts on the right show one example version of the emotion evaluation SIAT in detail

Both SIATs included one congruent and one incongruent association phase in separate sessions in randomized order. Prior to the first association phase, participants performed two shorter learning phases, where the assignment of keys for the categories was learned according to the first association phase. A learning phase consisted of either visual or auditory stimuli only. As an example, a congruent association phase was always preceded by two learning phases assigning auditory and visual emotions in a congruent fashion (e.g., happy voice = left, angry voice = right for the auditory learning phase and happy face = left and angry face = right for the visual learning phase in the emotion SIAT). The two association phases were separated by an additional re‐learning phase (either auditory or visual) with the identical setup, but with switched assignments of keys, preparing for the second association phase (see Figure 1 for a depiction of the experimental setup).

The order of the SIAT variants (emotion/gender) was randomized for each participant. The same was true for the order of the association phases within each SIAT (congruent/incongruent), the order of the learning phases (visual/auditory), and the assignment of emotion (angry/happy) and gender (male/female) to the response keys (right/left).

In the SIAT, reaction time differences between the congruent and incongruent association tasks quantified the implicit association of auditory and visual representations of the social categories emotion and gender. Both SIATs were conducted in a repeated measurement design on two different days. Auditory and visual stimuli were identical in both SIATs.

Although the original IAT has traditionally been used to measure attitudes (stereotypes/implicit bias) in social psychology (e.g., Gawronski, 2002; Wilson & Scior, 2013), research has shown that adaptations of the IAT paradigm can be used for associations between non‐social categories as well (e.g., flowers/insects and their association with pleasant/unpleasant attributes; Greenwald et al., 1998). Moreover, the IAT works for the auditory domain as well (McKay, Arciuli, Atkinson, Bennett, & Pheils, 2010) and even for the association between auditory and visual domains (Parise & Spence, 2012). This universal applicability encouraged us to use the SIAT as a social variant for investigating associations between vocal and facial stimuli.

In summary, the SIAT design allowed us to investigate the top‐down contributions of attentional focus and memory representation independently from each other. By using conjunction analyses, we were moreover able to identify activation patterns that were independent from stimulus modality (i.e., supramodal). To avoid any bias arising from the stimulus material itself (and thus to exclude any bottom‐up effects), we used exactly the same stimulus material for all association tasks.

Images were presented through a mirror mounted on the head coil. During the fMRI measurements, participants wore soft foam ear plugs and head phones, which served as ear protection as well as for delivering the auditory stimuli. The sound volume was tested before the measurements in the scanner and individually adjusted to a comfortable level, based on the participant's feedback. Previous experience with the same scanner, ear protection, and auditory stimulus set (e.g., Klasen et al., 2011) indicated that the stimuli were well audible and could easily be classified even with the scanner noise in the background. Responses were given via two keys on a keypad placed at the participant's right hand.

### 2.4 Data acquisition

Whole‐brain fMRI was conducted with echo‐planar imaging (EPI) sequences (TE = 28 ms, TR = 2,000 ms, flip angle = 77°, voxel size = 3 × 3 mm, matrix size = 64 × 64, 34 transverse slices, 3 mm slice thickness, 0.75 mm gap) on a 3 Tesla Siemens Prisma MRI scanner (Siemens Medical, Erlangen, Germany) using a 12‐channel head coil. The learning phases comprised 110 volumes each; association phases comprised 390 volumes. After the functional measurements, high‐resolution T1‐weighted anatomical images were performed using a magnetization prepared rapid acquisition gradient echo (MPRAGE) sequence (TE = 2.52 ms; TR = 1,900 ms; TI = 900 ms; flip angle = 9°; FOV = 256 × 256 mm^2^; 1 mm isotropic voxels; 176 sagittal slices). Total time for functional and anatomical scans was 45 min.

### 2.5 Data analysis

Image analyses were performed with BrainVoyager QX 2.8 (Brain Innovation, Maastricht, The Netherlands). Preprocessing of the functional MR images included slice time correction, 3D motion correction, Gaussian spatial smoothing (6 mm full width half maximum kernel), and high‐pass filtering including linear trend removal. The first five images of each functional run were discarded to avoid T1 saturation effects. Functional images were coregistered to 3D anatomical data and transformed into Talairach space (Talairach & Tournoux, 1988), following the standard procedure as implemented in BrainVoyager. In total, four participants were excluded from the analysis. One was excluded due to technical problems; a part of the original DICOM image files was damaged and could not be restored. Three additional participants were excluded from all further analyses due to excessive head motion as identified by visual inspection, leaving a total of 41 participants in the final sample. From the excluded participants, two were male and two were female, leaving a final sample of 21 female and 20 male participants.

Statistical parametric maps were created by using a random effects general linear model (RFX‐GLM) with multiple predictors according to the stimulus types. The following within‐subject factors were considered in the analysis:

Attentional focus (**Em**otion vs. **Ge**nder)
Congruency of target categories (**Co**ngruent vs. **In**congruent)
Stimulus modality (**Fa**ce vs. **Vo**ice)
Stimulus Gender (**Fe**male vs. **Ma**le)
Stimulus Emotion (**Ha**ppy vs. **An**gry)

The full combination of these five factors led to a total of 2^5^ = 32 predictors which are listed in Table 1 (abbreviations see above). For each of the contrasts, their encoding is marked with “+” and “−,” respectively.

**TABLE 1:** Predictors and their encoding

Predictor name	Figure 2a Blue	Figure 2a Red	Figure 2b Green	Figure 2b Yellow	Figure 4 Emotion	Figure 4 Gender	Figure 5 Blue	Figure 5 Red
EmCoFaFeHa		+	+		+			
EmCoFaMaHa		+	+		+			
EmCoFaFeAn		+	+		+			
EmCoFaMaAn		+	+		+			
EmCoVoFeHa	+		+		+			
EmCoVoMaHa	+		+		+			
EmCoVoFeAn	+		+		+			
EmCoVoMaAn	+		+		+			
EmInFaFeHa		+		+	−			
EmInFaMaHa		+		+	−			
EmInFaFeAn		+		+	−			
EmInFaMaAn		+		+	−			
EmInVoFeHa	+			+	−			
EmInVoMaHa	+			+	−			
EmInVoFeAn	+			+	−			
EmInVoMaAn	+			+	−			
GeCoFaFeHa		−	−			+		+
GeCoFaMaHa		−	−			+		+
GeCoFaFeAn		−	−			+		+
GeCoFaMaAn		−	−			+		+
GeCoVoFeHa	−		−			+	+	
GeCoVoMaHa	−		−			+	+	
GeCoVoFeAn	−		−			+	+	
GeCoVoMaAn	−		−			+	+	
GeInFaFeHa		−		−		−		−
GeInFaMaHa		−		−		−		−
GeInFaFeAn		−		−		−		−
GeInFaMaAn		−		−		−		−
GeInVoFeHa	−			−		−	−	
GeInVoMaHa	−			−		−	−	
GeInVoFeAn	−			−		−	−	
GeInVoMaAn	−			−		−	−	

Fixation cross phases served as a low‐level baseline. Events were defined in a stimulus‐bound fashion, that is, modeled for the duration of stimulus presentation. Only trials with correct responses were included in the analyses. Trials with missing or incorrect responses were modeled as separate confound predictors. Task contrasts were investigated via paired t tests. Following the recommendations of Woo, Krishnan, and Wager (2014), activations were thresholded at voxel‐wise *p* < .001 and Monte‐Carlo‐corrected for multiple comparisons on the cluster level (*p* < .05, corresponding to *k* > 11). All reported conjunction analyses tested the conservative conjunction null hypotheses (Nichols, Brett, Andersson, Wager, & Poline, 2005). Results are displayed in radiological convention (left is right).

To address the study's hypotheses, the following comparisons were of interest:

1. *Emotion versus gender: Supramodal networks*. These were networks specific for emotion resp. gender evaluation, but independent from stimulus modality. These were the contrasts (Voice Emotion > Voice Gender) ∩ (Face Emotion > Face Gender) as well as the reversed contrast (Gender > Emotion, respectively).
2. *Emotion versus gender: Networks independent from congruency of target categories*. These were networks specific for emotion resp. gender evaluation, but independent from congruency of the target categories. These were the contrasts (Congruent Emotion > Congruent Gender) ∩ (Incongruent Emotion > Incongruent Gender) as well as the reversed contrast (Gender > Emotion, respectively).
3. *Emotion versus gender: Networks depending exclusively on attentional focus*. These were networks depending solely on the attention focus (emotion or gender), independent from stimulus modality or congruency of the target concepts. This equals to the fourfold conjunction (Voice Emotion > Voice Gender) ∩ (Face Emotion > Face Gender) ∩ (Congruent Emotion > Congruent Gender) ∩ (Incongruent Emotion > Incongruent Gender) as well as the reversed contrast (Gender > Emotion, respectively).
4. *Networks depending on the congruency of target categories*. These were networks that were specific for congruency resp. incongruency of the target categories (congruent vs. incongruent and vice versa). They were investigated separately for emotion and gender evaluation, as well as for the comparison between them.
5. *Networks of supramodal memory representations*. Networks independent from stimulus modality, but depending on the congruency of the target concepts. This corresponds to the conjunction (Voice Congruent > Voice Incongruent) ∩ (Face Congruent > Face Incongruent), calculated for both emotion and gender classification.

## 3 RESULTS

### 3.1 Behavioral results

For emotion evaluation, 92.38% (118.24 ± 5.82) of all stimuli were classified correctly when target concepts were congruent, whereas 90.15% (115.39 ± 6.34) were classified correctly when target concepts were incongruent. Average reaction times for emotion evaluation were 985.90 ± 145.55 ms (congruent) and 1,150.01 ± 186.80 ms (incongruent). For gender evaluation, 95.26% (121.93 ± 5.38) of all stimuli were classified correctly when target concepts were congruent, whereas 94.40% (120.83 ± 4.92) were classified correctly when target concepts were incongruent. Average reaction times for gender evaluation were 859.63 ± 134.28 ms (congruent) and 1,012.68 ± 159.25 ms (incongruent).

In the SIAT—as well as in the original IAT—the Implicit Association Effect is quantified via reaction time differences between different pairings of target concepts. Increased reaction times reflect weaker associations between the investigated concepts and thus higher task difficulty. To investigate effects of target congruency, evaluation task, and their interaction on reaction times, we therefore calculated a 2 × 2 ANOVA with the factors Task (emotion evaluation/gender evaluation) and Congruency of target concepts (congruent/incongruent). Results revealed significant main effects of Task (*F*[1, 40] = 69.35, *p* < .001) and Congruency of target concepts (*F*[1, 40] = 174.96, *p* < .001), but no interaction (*F*[1, 40] = 0.40, *p* = .51).

RTs for the four association conditions (Emotion evaluation congruent, Emotion evaluation incongruent, Gender evaluation congruent, Gender evaluation incongruent) were tested for normal distribution. In all four conditions, reaction times were normally distributed (Kolmogorov–Smirnov test, *p*(EmoCong) = .20, *p*(EmoInkong) = .14, *p*(GendCong) = .20, *p*(GendInkong) = .16). Further data trimming was not performed. RTs and their *SD* in the present study were highly congruent with the data from a pre‐study on 42 subjects with very similar demographics without fMRI, using the exactly same paradigm. The comparison of the two data sets thus suggests a high level of replicability of the results.

### 3.2 Neuroimaging results

#### 3.2.1 Emotion versus gender: Supramodal networks

These were networks specific for emotion resp. gender evaluation, but independent from stimulus modality. We thus compared emotion versus gender evaluation networks separately for auditory (voice) and visual (face) stimuli.

**FIGURE 2:**
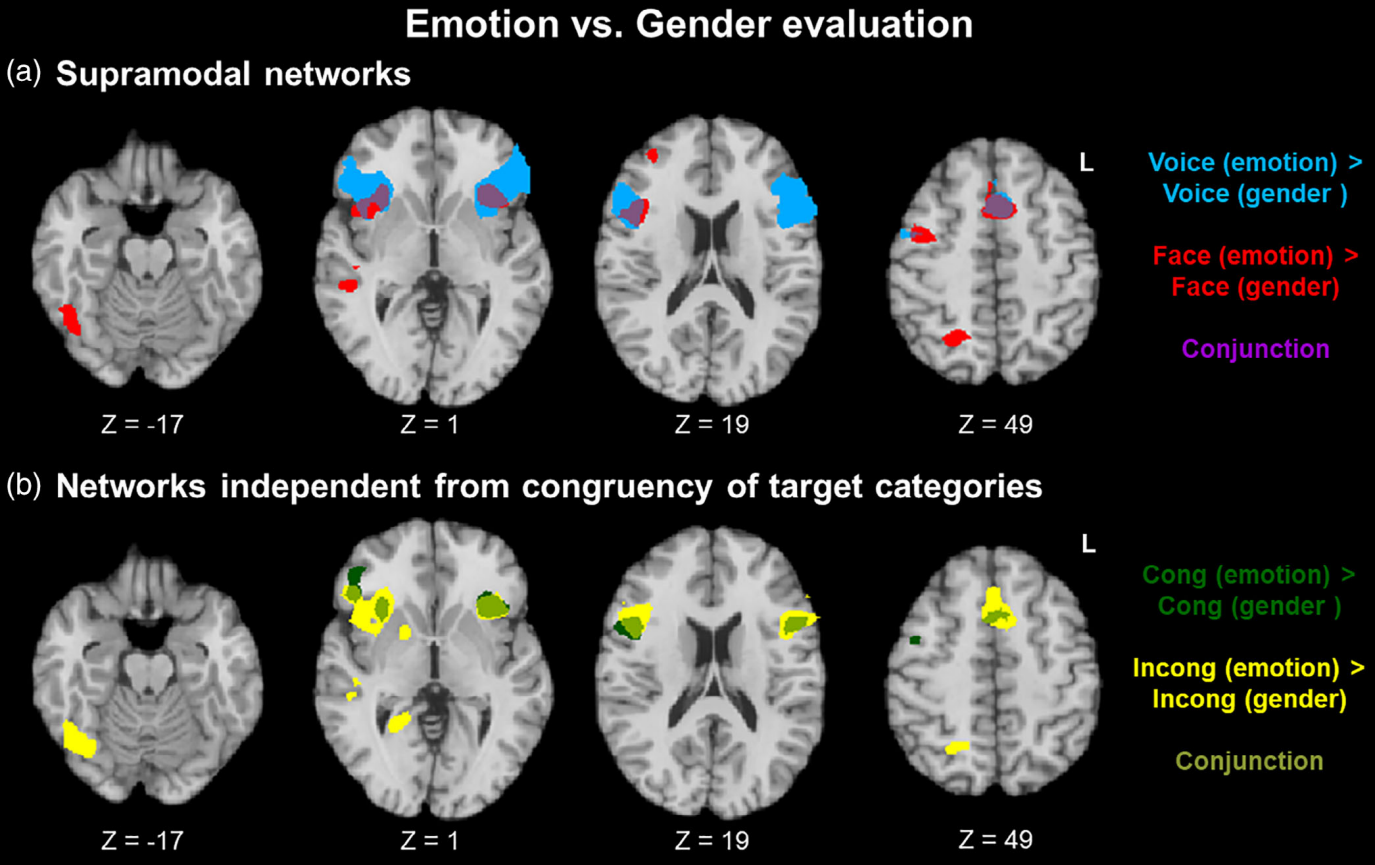
Emotion versus gender evaluation. (a) Supramodal networks. Besides some modality‐specific patterns such as fusiform face area for facial stimuli (red), emotion evaluation—as compared to gender evaluation—invoked a modality‐independent network in bilateral anterior insula, right inferior frontal gyrus (IFG), and pre‐supplementary motor area (SMA) (a; purple). A similar emotion evaluation network emerged independently from the congruency of the target categories (b; light green)

*Emotion > gender*. For emotion evaluation, auditory stimuli involved inferior frontal areas, along with pre‐supplementary motor area (pre‐SMA) and anterior insula (Figure 2a; blue). Visual stimuli enhanced activation in right fusiform face area (FFA) and superior temporal sulcus (STS), along with right inferior frontal gyrus (IFG) and middle frontal gyrus (MFG), pre‐SMA, and anterior insula (Figure 2a; red). A conjunction of both maps was observed in bilateral anterior insula, right IFG, and pre‐SMA (Figure 2a; purple). These areas thus reflect networks for emotion evaluation that are independent from stimulus modality.

*Gender > emotion*. For gender evaluation, auditory stimuli involved VMPFC and ACC, along with left angular and superior frontal gyri (Figure S1). No clusters emerged for visual stimuli or for the conjunction of both contrasts.

#### 3.2.2 Emotion versus gender: Networks independent from congruency of target categories

These were networks specific for emotion resp. gender evaluation, but independent from congruency of the target categories.

*Emotion > gender*. For emotion evaluation, the congruent condition involved bilateral IFG, pre‐SMA, and bilateral anterior insula (Figure 2b; dark green), whereas the incongruent condition enhanced activation in globus pallidus, right FFA and STS, bilateral IFG, pre‐SMA, and bilateral anterior insula (Figure 2b; yellow). A conjunction of both maps was observed in bilateral anterior insula, right IFG, and pre‐SMA (Figure 2b; light green). These areas thus reflect networks for emotion evaluation that are independent from the congruency of target concepts.

*Gender > emotion*. For gender evaluation, congruent target categories involved left angular gyrus (Figure S2). No clusters emerged for incongruent target categories or for the conjunction of both contrasts.

#### 3.2.3 Emotion versus gender: Networks depending exclusively on attentional focus

These were networks depending solely on the attention focus (emotion or gender), independent from stimulus modality or congruency of the target concepts.

**FIGURE 3:**
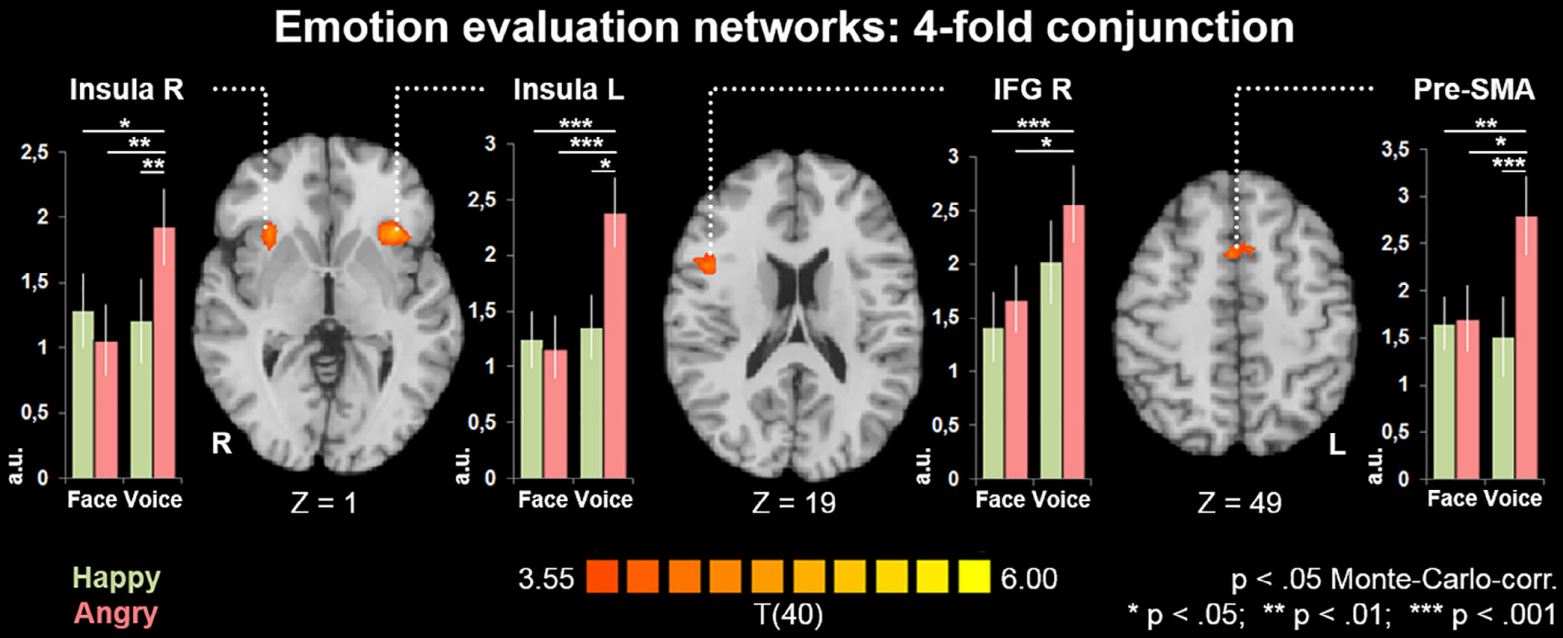
Emotion evaluation networks: fourfold conjunction. The fourfold conjunction (Voice emotion > Voice gender) ∩ (Face emotion > Face gender) ∩ (Congruent emotion > Congruent gender) ∩ (Incongruent emotion > Incongruent gender) confirmed bilateral anterior insula, right inferior frontal gyrus (IFG), and pre‐supplementary motor area (SMA) as an emotion evaluation network irrespective of stimulus modality and congruency of target concepts. This network thus reflects the top‐down contribution of attentional focus during emotion evaluation. In all regions of this network, angry voices evoked the strongest response (a.u. = arbitrary units, derived from mean beta values from the first level general linear models [GLMs]). No such attention‐driven network was observed for gender evaluation

*Emotion > gender*. To investigate effects specific for emotion evaluation independently from stimulus modality and from the congruency of target concepts, we thus calculated the fourfold conjunction of all maps, that is, (Voice emotion > Voice gender) ∩ (Face emotion > Face gender) ∩ (Congruent emotion > Congruent gender) ∩ (Incongruent emotion > Incongruent gender). The resulting map revealed a common activation in bilateral anterior insula, right IFG, and pre‐SMA (Figure 3; Table 1).

Differences in reaction times between emotion and gender evaluation indicated a higher difficulty of the emotion task. To investigate possible influences of the latter on the brain activation patterns displayed in Figure 3, we calculated a subject‐wise task difficulty coefficient for this contrast, which was defined as (AverageRTEmotion − AverageRTGender). This coefficient was correlated with individual contrast values in all clusters. Even at a liberal threshold of *p* < .05 and without correction for multiple comparisons, no correlation with task difficulty was observed in any of the clusters (IFG R: *r*(39) = .01, *p* = .94; Ant Ins R: *r*(39) = −.16, *p* = .31; SMA: *r*(39) = −.25, *p* = .12; Ant Ins L: *r*(39) = −.04, *p* = .80). For the regions of this network, we moreover compared contrast values between different stimulus types with paired t tests (Figure 3). Notably, angry voices constantly yielded the strongest effect in all four regions, whereas no difference was observed between any of the other three stimulus types (happy voice, angry face, happy face).

*Gender > emotion*. No clusters emerged for the fourfold conjunction (Voice gender > Voice emotion) ∩ (Face gender > Face emotion) ∩ (Congruent gender > Congruent emotion) ∩ (Incongruent gender > Incongruent emotion).

#### 3.2.4 Networks depending on the congruency of target categories

These were networks that were specific for congruency resp. incongruency of the target categories (congruent vs. incongruent and vice versa).

**FIGURE 4:**
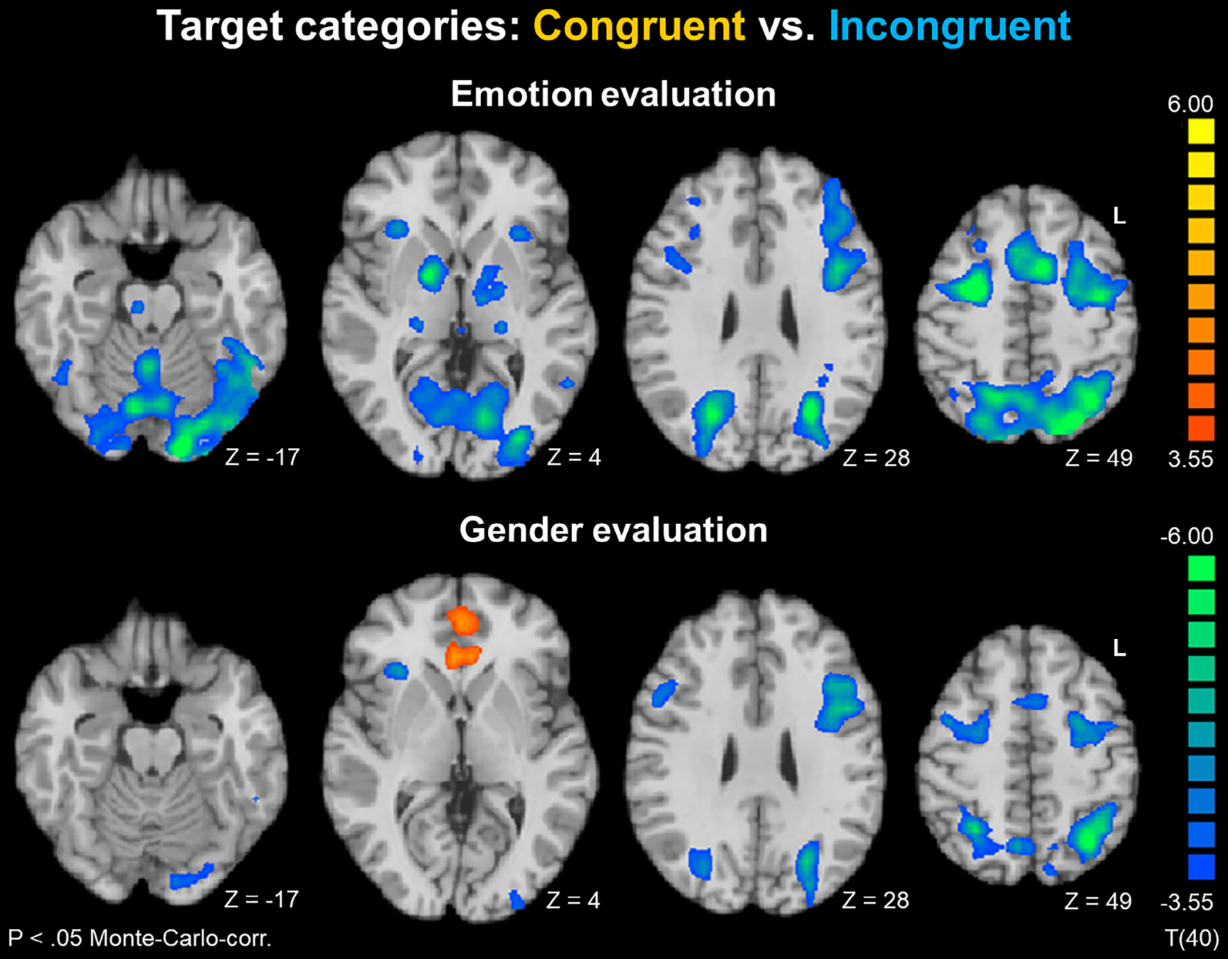
Target categories: Congruent versus incongruent. Both emotion and gender evaluation showed similar fronto‐parietal networks for incongruent target categories, indicating increased working memory load. No congruency‐specific activation was observed for emotion evaluation. For gender evaluation, congruency led to enhanced activation in rostral anterior cingulate cortex (rACC) and ventromedial prefrontal cortex (VMPFC). These networks may thus reflect supramodal memory representations supporting gender evaluation

*Emotion evaluation*. Incongruence led to a stronger activation in areas of the emotion evaluation network (compare Figure 2), namely FFA, bilateral anterior insula, thalamus, globus pallidus, IFG/MFG, and pre‐SMA, along with extended activation in visual systems, DLPFC, and superior parietal lobule (SPL; Figure 4). Congruency, in contrast, was not associated with any specific activation pattern during emotion evaluation.

*Gender evaluation*. A different picture emerged for the gender evaluation task. The incongruent condition led to a similar, albeit less pronounced pattern in pre‐SMA, DLPFC, right anterior insula, and SPL. Congruency, in turn, led to enhanced activation in two prominent clusters in rACC and VMPFC (Figure 4).

#### 3.2.5 Networks of supramodal memory representations

These were networks independent from stimulus modality, but depending on the congruency of the target concepts.

*Emotion evaluation*. No clusters emerged for the conjunction (Voice congruent > Voice incongruent) ∩ (Face congruent > Face incongruent).

**FIGURE 5:**
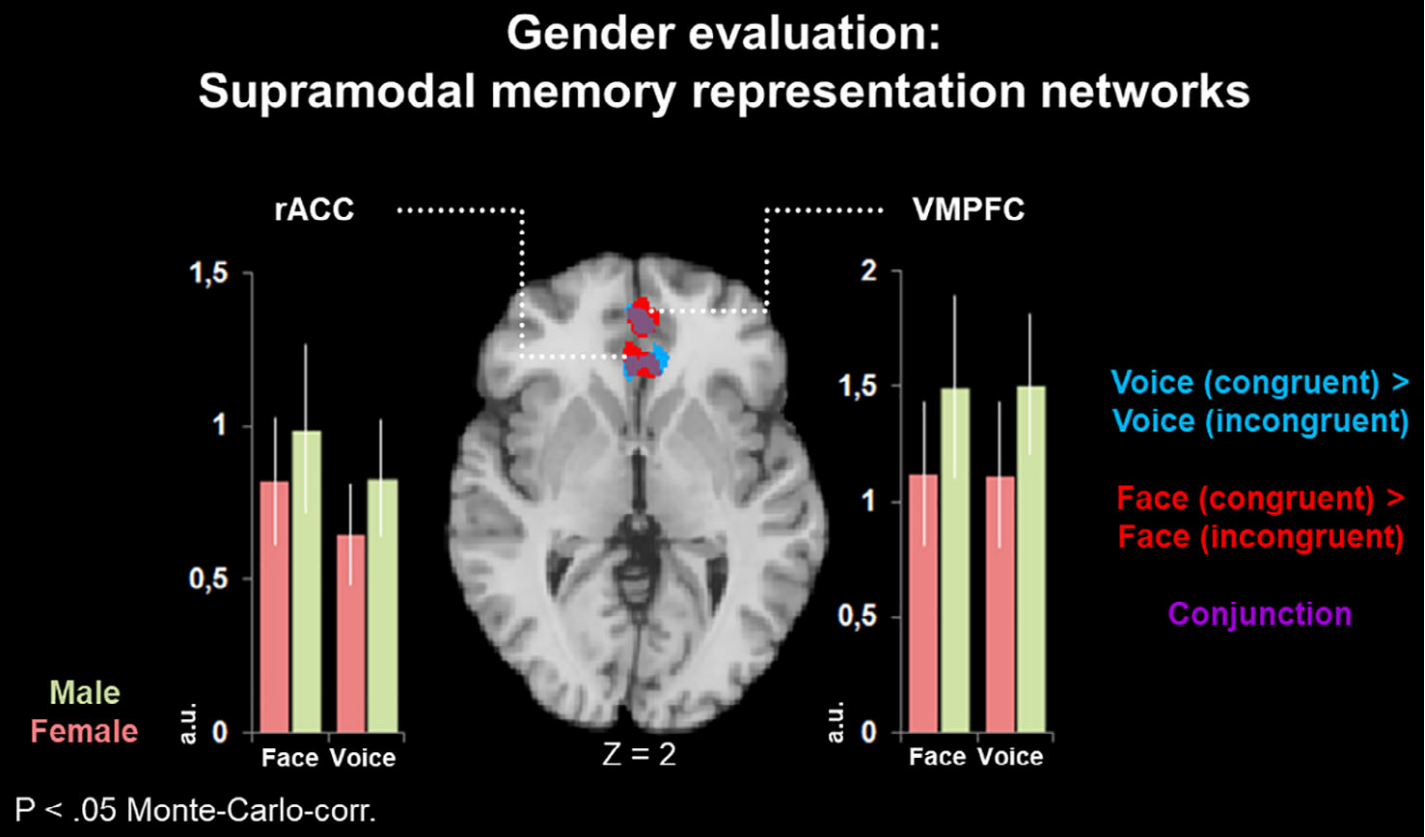
Gender evaluation: Supramodal memory representation networks. For gender evaluation, congruency‐specific networks in rostral anterior cingulate cortex (rACC) and ventromedial prefrontal cortex (VMPFC) were independent from stimulus modality (conjunction of Voice and Face; purple), thus confirming the contribution of a supramodal memory network. Within this network, no significant differences emerged between male and female stimuli (a.u. = arbitrary units, derived from mean beta values from the first level general linear models [GLMs]). For emotion evaluation, no such network was observed

*Gender evaluation*. The maps for auditory (blue) and visual stimuli (red) largely overlapped in both clusters (Figure 5), as reflected by the conjunction (Voice congruent > Voice incongruent) ∩ (Face congruent > Face incongruent), displayed in purple. In both regions, contrast values were compared with paired t tests. Within each region, no differences emerged between different evaluated stimulus types (male and female faces and voices). The clusters from the functional maps in Figures 2, 3, 4, 5 are listed in Table 2.

## 4 DISCUSSION

The present study revealed new insights into top‐down contributions to social perception. Specifically, the SIAT enabled us to identify top‐down influences in the processing of social information in the auditory and visual domains. Task‐specific, but modality‐independent patterns reflected supramodal networks for social categories. These top‐down components could further be separated into attention‐driven networks and supramodal memory representations. Functionally distinct networks were identified for the social categories emotion and gender.

For emotion evaluation, the modality‐specific analysis revealed FFA and STS specifically for face processing. Besides the FFA's well‐established role in face identification, which is assumed to rely on mainly invariant facial features (Calder & Young, 2005; Dekowska, Kuniecki, & Jaśkowski, 2008; Haxby, Hoffman, & Gobbini, 2002; Hoffman & Haxby, 2000), recent studies highlight the importance of the FFA for processing emotional expressions as well (Harry, Williams, Davis, & Kim, 2013; Nestor, Plaut, & Behrmann, 2011). In line with these findings, our study supports the notion of the FFA as part of a visual emotion recognition network. Moreover, we extend present findings by demonstrating a task dependency of the stimulus‐evoked activation. Specifically, FFA activity to facial stimuli was observed only during emotion, but not during gender classification, therefore reflecting top‐down modulation. Evaluative networks in the FFA thus seem to rely on the attentional focus.

Moreover, we confirmed the role of the posterior right STS for conscious processing of visual emotions. The STS was traditionally regarded as a flexible component in different neural pathways without a specialized functional attribution (for a review see Hein & Knight, 2008). Indisputably, the STS has various functions including Theory of Mind abilities, social perception, and multisensory integration (Amedi, von Kriegstein, van Atteveldt, Beauchamp, & Naumer, 2005; Campanella & Belin, 2007; Saxe, 2006). This functional versatility may be explained by sub‐regional specialization, but also by task‐dependent co‐activation with functionally distinct frontal and temporal networks (Hein & Knight, 2008). Functional synchronicity of posterior STS with the FFA may reflect a visual emotion processing network.

Current models of auditory emotion processing highlight a right‐hemispheric lateralization (Brück, Kreifelts, & Wildgruber, 2011; Klasen et al., 2018). Right sided primary and higher order acoustic regions extract suprasegmental information, followed by processing of meaningful suprasegmental sequences in posterior parts of the right STS, followed by evaluation of emotional prosody in IFG (Wildgruber, Ackermann, Kreifelts, & Ethofer, 2006). Neuroimaging findings (Klasen et al., 2018) highlight the relevance of right IFG for emotional prosody. In our study, a right‐hemispheric lateralization was observed for the fourfold conjunction of all maps (Figure 3c), showing specific effects of emotion evaluation independently from modality and congruency of target concepts. Extending previous findings, our study highlighted the role of right IFG in emotion recognition from both auditory and visual domains. From an integrative perspective, there may thus be a supramodal right hemispheric dominance for emotion processing (cf. Le Grand, Mondloch, Maurer, & Brent, 2003).

Functional mapping of explicit emotion processing furthermore revealed effects in bilateral anterior insula and pre‐SMA. Research has shown that the pre‐SMA is involved in domain‐general sequence processes (Cona & Semenza, 2017) and in emotional evaluation of signals irrespective of modality (Ethofer et al., 2013). The anterior insula has been ascribed to a wide range of complex functions and participates in various cognitive and emotional processes (for a review see Menon & Uddin, 2010). In line with our findings, Menon and Uddin (2010) propose that a basic function of the anterior insula is the bottom‐up driven detection of salient stimuli across multiple modalities. It is well established that the insula engages in affective processes (e.g., emotion perception of others) and the experience of emotions that derive from visceral and somatic information about bodily states (Uddin, 2015). As such, insula activity represents an individual's subjective and conscious emotional state, as well as the emotive value of external stimuli. Thus, it has been suggested that the ability to understand the emotions of others depends largely on experiencing similar changes in our visceral state by mirroring the perceived emotion (Critchley & Harrison, 2013). The anterior insula may be a central hub in this function. Taken together, the observed activity of SMA and anterior Insula may represent a supramodal neuronal signature of explicit emotion processing. However, since similar patterns emerged for the congruent as well as for the incongruent target concept they reflect an evaluative rather than a supramodal memory network. Considering the similarity with the salience network (Menon, 2015), the pattern reflects the high evolutionary significance of emotion recognition.

**TABLE 2:** Clusters from mapping in Figures 2, 3, 4, 5

Cluster	Brain region	TAL coordinates				
		x	y	z	Peak T	mm^3^
Figure 2a: conjunction voice and face						
1	Inferior frontal gyrus r	42	20	16	4.95	3,048
2	Insula r	30	17	1	4.61	1,563
3	Pre‐supplementary motor area r/l	−6	11	49	5.49	2,051
4	Insula l	−33	20	1	5.28	1,961
Figure 2b: Conjunction congruent and incongruent						
1	Inferior frontal gyrus r	45	11	16	4.56	1,738
2	Insula r	30	20	1	4.78	544
3	Pre‐supplementary motor area r/l	6	11	61	4.53	821
4	Insula l	−33	20	1	5.50	1,978
5	Inferior frontal gyrus l	−45	14	19	5.26	630
Figure 3: Fourfold conjunction						
1	Inferior frontal gyrus r	45	11	16	4.56	1,010
2	Insula r	30	17	1	4.45	510
3	Pre‐supplementary motor area r/l	6	11	61	4.53	808
4	Insula l	−33	20	1	5.28	1,564
Figure 4: Congruent > incongruent target categories						
Emotion						
1	Inferior frontal gyrus r, Insula r	30	23	7	−5.32	4,165
2	Fusiform gyrus r	45	−58	17	−4.19	297
3	Inferior parietal lobule r/l, superior parietal lobule l/r, Cuneus r/l, lingual gyrus r/l, inferior/middle occipital gyrus r/l, fusiform gyrus l, brainstem r/l, thalamus r/l, Cerebellum r/l	27	−61	−17	−8.74	133,612
4	Precentral gyrus r, superior frontal gyrus r, middle frontal gyrus r	27	−4	49	−7.89	8,409
5	Superior frontal gyrus r, middle frontal gyrus r	27	23	43	−4.66	2,384
6	Thalamus r, Globus pallidus r	15	−4	1	−6.76	4,717
7	Inferior frontal gyrus l, Insula l, precentral gyrus r, superior frontal gyrus r, middle frontal gyrus r, pre‐supplementary motor area r/l	−33	−4	58	−7.20	32,879
8	Thalamus r, Globus pallidus r	−21	−22	−2	−5.23	3,392
Gender						
1	Precentral gyrus r, superior frontal gyrus r, middle frontal gyrus r	36	−4	43	−5.07	5,084
2	Insula r	36	−13	19	−4.33	441
3	Insula r	27	23	7	−4.94	535
4	Superior parietal lobule r/l, inferior parietal lobule l/r, Precuneus l/r	−30	−55	43	−8.11	28,037
5	Cerebellum r/l	9	−70	−26	−4.87	1,251
6	Precentral gyrus l, superior frontal gyrus l, middle frontal gyrus l	−42	2	37	−7.57	12,501
7	Ventromedial prefrontal cortex r/l	0	47	4	4.76	2,735
8	Rostral anterior cingulate gyrus r/l,	0	32	−2	4.99	2,405
9	Cuneus l, lingual gyrus l	−12	−88	−14	−5.00	3,570
10	Fusiform gyrus l	−45	−52	−11	−4.48	836
Figure 5: Conjunction voice and face						
1	Ventromedial prefrontal cortex r/l	0	47	1	3.98	736
2	Rostral anterior cingulate cortex r/l	−3	32	−2	4.27	566

Notably, angry voices elicited the strongest responses in the emotion evaluation network. Previous research revealed an overall increase in activation for vocal emotion compared with neutral expressions in a fronto‐temporo‐striatal network (Ethofer et al., 2006; Kotz et al., 2003). Ethofer et al. (2009) investigated brain regions that were more responsive to angry than to neutral prosody and identified bilaterally IFG/OFC, amygdala, insula, mediodorsal thalamus, and the middle part of the STG. Furthermore, they showed that the activation of these regions was automatic and independent of the underlying task, concluding that angry prosody is processed irrespectively of cognitive demands and attentional focus. Similar findings can be observed for visual emotion processing. Vuilleumier (2005) inferred that the FFA was more activated by fearful than neutral faces, even when faces were task‐irrelevant. Our findings support the notion that angry prosody is perceived with particular dominance, which is of fundamental importance to prioritize the procession of threat‐related stimuli (Cox & Harrison, 2008; LeDoux, 2003).

Remarkably, no amygdala activation was observed for any of the emotion classification categories. Lesion studies show that the amygdala has modulatory influences on emotion processing areas and heightens activity in for example, the FFA when perceiving fearful faces compared to neutral (Vuilleumier, Richardson, Armony, Driver, & Dolan, 2004), and this is also true for prosodic emotion processing and the STS (Frühholz et al., 2015). Since emotional information was present in all trials, missing amygdala differences can be attributed to unattentional emotion processing in all tasks. This notion is supported by previous findings (Vuilleumier, 2005; Vuilleumier, Armony, Driver, & Dolan, 2001) and was also explicitly validated in trials with a gender classification task, where amygdala activation was present even though attention was directed to the gender (Morris, Ohman, & Dolan, 1998).

A comparison of congruent versus incongruent target concepts revealed increased workload in a fronto‐parietal network for incongruence in emotion and gender SIATs. This network has already been described for the evaluation of semantic incongruent bimodal emotional stimuli (Klasen et al., 2011). It shows a large overlap with the executive control network as described by Seeley et al. (2007), which reflects attention, working memory, and response selection. Almost identical findings between our study and Klasen et al. (2011) indicate a negligible influence of stimulus modality. Instead, the network seems to be driven by the aspect of incongruence itself, putatively reflecting increased task difficulty and cognitive workload in the incongruent condition. Moreover, pre‐SMA activity may reflect conflict monitoring and error detection (Mayer et al., 2012). In a similar way, social classification categories (emotion vs. gender) seem to be only of minor importance for incongruence networks.

In contrast to our initial hypotheses, we could not reveal a contribution of a supramodal memory representation for emotional categorization. Instead, emotion evaluation seems to involve large evaluative networks, some of them modality‐independent, others not. In summary, recognition of facial and vocal emotions involves common networks in insula, IFG, and pre‐SMA, but does not rely on a common supramodal memory representation. In line with this notion, a recent meta‐analysis by Schirmer (2018) revealed fundamentally different pathways for auditory and visual emotions. Effects of effortful, that is, conscious emotion processing were observed in supplementary motor regions, which is in line with our findings. Emotion processing effects in limbic areas such as the amygdala, in turn, were task‐independent and largely driven by the visual modality (Schirmer, 2018). This also delivers a new perspective on crossmodal emotion integration. Neuroimaging findings show that congruent audiovisual emotions enhance activity primarily in limbic areas (Klasen et al., 2011). In line with the well‐established visual dominance effect in audiovisual perception (Colavita, 1974), auditory emotions may be just a supplement to visual perception, both behaviorally and neurobiologically, without the need for recruiting a common memory representation.

The gender SIAT, in contrast, showed enhanced involvement of two prominent clusters: the VMPFC and ACC. These findings are well in line with the findings from Knutson et al. (2007) on the neural substrates of gender and racial bias, as well as with Milne and Grafman (2001), who found a reduced IAT effect in patients with VMPFC lesions. In these studies, VMPFC was considered as representing previously learned automatic processing of emotional and social information. Thus, VMPFC may support concept formation in long‐term memory.

Widening the scope, this view is very much in line with neuroimaging research on schematic memory. Schemas are experience‐based implicit memory representations of situational aspects that typically belong together. They are activated by perceptual input and form a framework for stimulus interpretation (Bowman & Zeithamova, 2018; Spalding, Jones, Duff, Tranel, & Warren, 2015), a conceptualization closely related to the spreading activation network theory by Collins and Loftus (1975). Recent neuroimaging studies highlight VMPFC contributions to establishing and retrieving schemas. A lesion study by Spalding et al. (2015) indicated reduced performance ability of subjects with focal VMPFC damage for integrating new information into a schema congruent context. In a recent fMRI study, Bowman and Zeithamova (2018) describe the VMPFC as representing abstract prototype information, supporting generalization in conceptual learning over multiple domains. In summary, the VMPFC seems to store memories about typical examples and characteristic features of object categories. These “prototype” representations seem to facilitate object recognition in a top‐down fashion: classification and response selection are based on the comparison of perceptual input with memory prototypes. In the case of gender, facial and vocal stimuli seem to access the same supramodal memory prototype, which may also account for the enhanced accuracy and reaction times compared to the emotion classification task. Supramodal prototypes may exist for emotions as well; however, the present study found no evidence for their contribution to stimulus classification.

## 5 CONCLUSION

The present study identified modality‐specific and modality‐independent influences of attentional focus and memory representations on the neural processing of social stimuli. Irrespective of modality, emotion evaluation engaged a fronto‐insular network which was independent from supramodal memory representations. Gender classification, in turn, relied on supramodal memory representations in rACC and VMPFC.

## CONFLICT OF INTEREST

The authors declare no potential conflict of interest.

## Supporting information


**Figure S1** Supporting information

**Figure S2** Supporting information

## Data Availability

The data that support the findings of this study are available from RWTH Aachen University. Restrictions apply to the availability of these data, which were used under license for this study. Data are available from the corresponding author with the permission of RWTH Aachen University.
