# Supplementary figures and images for: Supramodal neural networks support top‐down processing of social signals
Source: Hum Brain Mapp. 2020 Oct 19;42(3):676–89. doi: 10.1002/hbm.25252 (PMC7814753; doi:10.1002/hbm.25252)

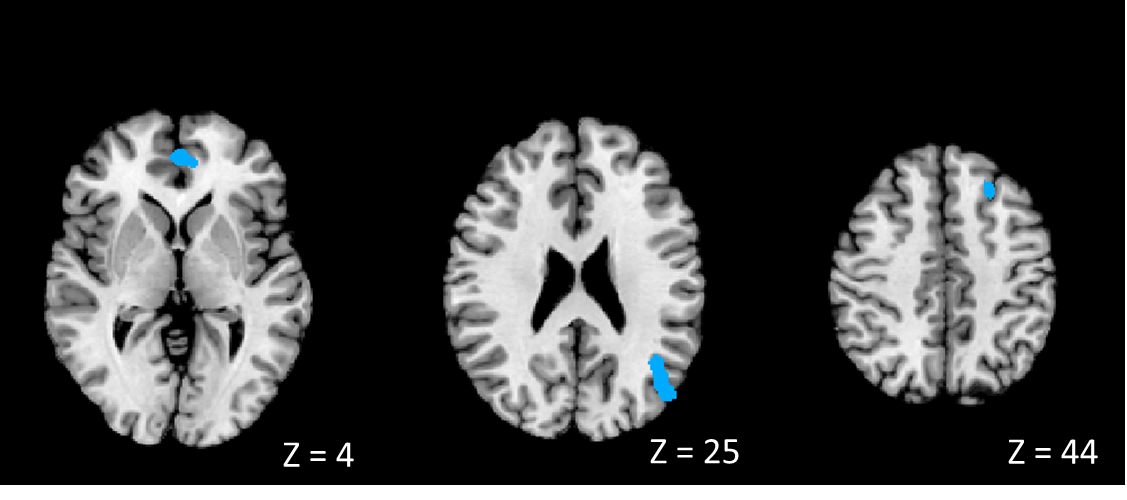

Supplement: Supplementary file 1 — Figure S1 Supporting information [file HBM-42-676-s001.tif]

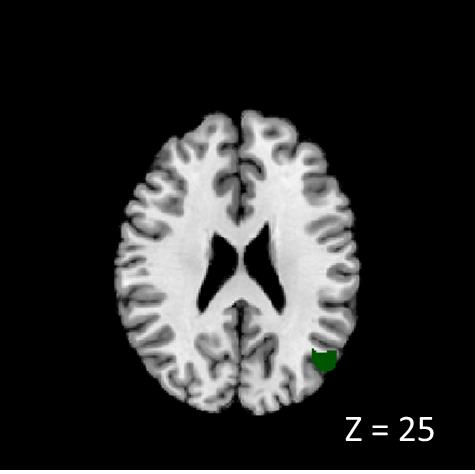

Supplement: Supplementary file 2 — Figure S2 Supporting information [file HBM-42-676-s002.tif]
